# Savoring life during pandemic: an online intervention to promote well-being in emerging adults

**DOI:** 10.1186/s40359-023-01225-z

**Published:** 2023-07-04

**Authors:** Daniela Villani, Elisa Pancini, Francesca Pesce, Lucia Scuzzarella

**Affiliations:** 1 Department of Psychology, Research Center in Communication Psychology (PsiCom), Università Cattolica del Sacro Cuore, Largo Gemelli, 1, 20123 Milan, Italy; 2 Department of Psychology, Università Cattolica del Sacro Cuore, Milan, Italy

**Keywords:** Savoring, Emerging Adults, Subjective Well-Being, Positive Emotions, Online Interventions, COVID-19 Pandemic

## Abstract

**Background:**

Savoring, that is the ability to create and increase positive emotions, represents a promising approach to enhance subjective well-being (SWB) in emerging adults. This controlled study aims to investigate the preliminary effects of a self-help e-savoring intervention on increasing savoring beliefs and strategies and SWB in times of the COVID-19 pandemic.

**Methods:**

Forty-nine emerging adult participants were recruited using the snowball sampling method. The experimental group (*n* = 23) completed six online exercises (two exercises per week for three weeks) while the control group (*n* = 26) did not receive the intervention. Both groups filled out online questionnaires before and after the intervention. User experience and perceived usefulness of the intervention were assessed for the experimental group.

**Results:**

A repeated measures analysis of variance (ANOVA) revealed a significant increase for the experimental group in savoring beliefs (especially toward the present and the future) and in positive emotions compared to the control group. The perspicuity, attractiveness, and efficiency of the online platform were very positively evaluated, and most participants rated the intervention as useful.

**Conclusions:**

The results of this preliminary study together with the high level of adherence and the appreciation for the intervention indicate the potential of promoting online savoring and positive emotions in emerging adults. Future research could evaluate its long-term effects and verify its results with other age groups.

## Background

The lives of people across the world have been severely impacted by the COVID-19 pandemic. Not only major health risks and economic uncertainty but also mental health challenges have been extensively documented [1]. Among the different populations that have been psychologically impacted by the COVID-19 crisis, emerging adults, such as individuals aged 18–30 years [2], who are already called on to face complex and uncertain challenges [3] (e.g., completing their education, leaving the parental home, finding a job, having a stable romantic relationship, and eventually having children), need to be seriously taken into consideration [4, 5].

COVID-19 containment measures such as social distancing, lockdowns, the closure of borders, businesses, and universities have been effective in slowing the spread of the virus and protecting physical health, but they have also limited emerging adults’ opportunities for independence, personal and professional growth, as well as interactions with family members, friends, and co-workers [6, 7]. In particular, all of these unexpected changes together with the impact of COVID itself (e.g., oneself or family members becoming seriously ill, or worries about it) have caused a burden of mental health conditions including anxiety, depression, irritability, reduced sleep quality, psychological distress over the uncertainty of the future, frustration, and loneliness [8–13], and have significantly decreased emerging adults’ subjective well-being (SWB) [14]. In fact, according to the ISTAT (Istituto Nazionale di Statistica) report of 2021, in Italy, both adolescents and emerging adults with ages ranging between 19 and 24 reported a decrease in SWB [15–17].

SWB is a combination of people’s overall evaluation of how satisfied they are with their lives and their affective responses to everyday events [18]. Several studies have shown that SWB, especially in the form of positive affect, is associated with protective psychological and behavioral factors such as greater social connection, greater perceived social support, more optimism, and a greater likelihood of engaging in healthy behaviors and correlates [18]. Indeed, SWB can foster better psychological health, and it is recognized as a critical factor in the prevention of depression [12, 19, 20]. Some uncontrollable life events and major unexpected changes, such as those associated with a pandemic, can undermine people's SWB, and for this reason developing interventions to support the subjective well-being of young adults in uncertain times is of relevant interest.

### Savoring and subjective well-being

Among positive psychology interventions (PPIs), one promising approach that already demonstrated its positive impact on SWB is savoring. Savoring is the ability to generate, prolong, or intensify positive emotions, and it can increase awareness and recognition of the latter [21–24]. Therefore, the essence of savoring lies in the “conscious awareness of ongoing positive feelings” related to the experience [25]. While savoring, people become aware of ongoing positive feelings, and the intensity and duration of these feelings change.

Theoretically, savoring has been identified as a mechanism strictly connected to the well-known theories of positive psychology, namely the Broaden and Build Theory of Positive Emotions and the Undoing Hypothesis [26–29]. Indeed, savoring allows the generation, maintenance, and amplification of positive feelings and buffers against adverse health outcomes and negative emotions [29, 30]. Savoring can involve a form of mental time journey through which individuals can generate positive emotional states in the present thanks the focus on the here and now (present moment), the imagination of past (positive reminiscence) or future positive (positive anticipation) events [22, 28].

Research has shown that people may or may not be aware of their savoring skills (savoring beliefs). According to Bryant [31], “savoring beliefs involve a conscious awareness of one’s ability or inability to experience and manage positive experience.” On the one hand, the evidence suggests the presence of individual differences in savoring beliefs that are associated with personal traits such as extraversion, optimism, and emotional stability [24, 32, 33]. On the other hand, Bryant and Veroff [24] identified several savoring strategies that can amplify the intensity and duration of positive emotions. They are, for example, sharing with others, such as experiencing a positive event with a close friend; memory building, which is taking mental photographs of an event to remember it; self-congratulation, such as focusing on personal life achievements; sensory-perceptual sharpening, which is grasping every sensory aspect of a positive event; feeling absorbed in the moment; and using behavioral expressions such as laughing or jumping. Finally, other strategies include having a temporal awareness of the experience (for example, remembering that events end and must be fully experienced when they occur), and counting blessings such as thinking of small positive things of the day.

A meta-analytical review conducted by Smith et al. [34] found a substantial positive impact of savoring interventions on the intensity and duration of positive emotions and on well-being. Savoring outperforms other interventions such as optimism in terms of increasing positive affect, happiness, and life satisfaction while decreasing negative affect [35].

Savoring has been positively associated with well-being and negatively associated with depressive and anhedonia symptoms in both youth and adults [21, 28]. Until now, only a few savoring interventions for emerging adults have been proposed, and they have shown promising results. Bryant and Veroff [24] asked college students to notice as many pleasant aspects as possible of the environment around them (e.g. the flowers, the sun) during a 20-min walk. A week later, these students reported higher levels of happiness than those belonging to the neutral condition and the negative focus condition. Other researchers trained 94 college students to exercise the savoring strategies through recorded or written instructions, and, after 2 weeks, they found a significant reduction in depression and negative affect [36].

### Online savoring

Recently, the broad approach of positive technology has recognized the positive role that technologies can have in promoting well-being by taking advantage of different strategies [37, 38]. Within this perspective, online positive psychology interventions (OPPIs) show several potentialities: they are accessible from different devices, they can reach different populations, and they are cost-effective [39–42]. Furthermore, OPPIs provide several advantages for emerging adults, as they are “digital natives” and use the Internet and apps daily for many activities and are favorable toward using self-help content [41, 43]. Digital psychological self-help interventions can be accessed from any location, thus overcoming logistical and geographical barriers to treatment delivery, and often resulting in more affordable and less time-consuming interventions in comparison to traditional interventions, with a subsequent significant improvement in self-monitoring processes [44–46].

Up to now, savoring has been integrated with other strategies in online interventions. This is the case with Heintzelman et al.’s [47, 48] 12-week in-person and online program for promoting SWB called "ENHANCE.". Participants, aged between 25 and 75, were asked to perform certain actions, including complimenting others, actively listening to other people, identifying and focusing on positive events, savoring, practicing mindfulness, and expressing gratitude. At the end of the intervention, participants belonging to both the in-person and online experimental groups reported higher levels of life satisfaction and positive affect and a reduction of negative affect, depressive symptoms, and perceived stress compared to the waiting list control group.

In other recent research, savoring interventions alone have been delivered and tested online. The pilot study by Park [49] aimed to teach the components of savoring through a website by providing several psychoeducational resources (articles, videos, and books) to promote the daily practice of savoring. The intervention lasted 21 days and showed the potential for an online self-managed savoring intervention to increase savoring and subjective well-being. However, only 22% of the initial participants (128) completed the intervention. Yu et al. [50] asked Taiwanese college students to perform a pleasant activity by paying attention to the positive emotions they felt for 20 min a day three times a week. Afterwards participants were asked to describe and post these emotions on a social network (e.g., Facebook) by using a photo and/or a text. The study found a significant increase in positive affect and decrease in depression in the experimental group in the post-test compared to the control condition. However, the decreased level of depression was not maintained in the follow-up.

### The present study

When people are overwhelmed by negative events, they may be unable to notice or appreciate positive things; in fact, threats, distress, or distraction can inhibit people’s ability to savor positive experiences [25]. Being able to recognize and appreciate positive moments during challenging events can be a source of strength [29, 51–53]. Thus, savoring can represent a favorable approach in dealing with challenges such as the COVID-19 pandemic.

Starting from these premises and taking advantage of the potential of a self-help approach [54], we developed a self-help e-savoring intervention (Savor Project) and conducted a preliminary test of its benefits for emerging adult participants. We hypothesized that (1) people in the experimental group would more strongly believe in their ability to enjoy positive events (savoring beliefs) compared to the control group. Concerning SWB, we hypothesized that (2) participants in the experimental group would have a higher level of life satisfaction and positive affect and a lower level of negative affect than participants in the control group. We also explored whether the intervention would increase the likelihood of behaviors and/or thoughts that can amplify the intensity and duration of positive emotions (savoring strategies). In addition, we categorized the qualitative experiences written as comments by participants in terms of elicited emotions, bodily awareness, and content of the visualized experiences. Finally, we evaluated the user experience and the perceived usefulness of the intervention.

## Methods

### Participants

The participants were recruited using the snowball method thanks to posters published on social media and through word of mouth. The sample size of the feasibility study was calculated with G Power [55], and it was found that 46 participants would be needed to achieve 90% power with an average effect size (*f* = 0.25) with a repeated measurement design within/between interaction (number of groups 2; number of measurements: 2; correlation between repeated measurements: 0.5). In the current study, 55 emerging Italian adults were randomly assigned to the experimental and the control groups using block randomization. A label of A or B (A = intervention, B = control) was assigned to each group (block size = 4). Free online software (Research Randomizer 4.0) was used to generate the randomization list. Six people belonging to the experimental condition were excluded from the final sample because they did not complete all the activities, resulting in final sample sizes of 23 in the experimental condition and 26 in the control condition. The only inclusion criteria were age between 18 and 30 years, fluency in the Italian language, and having Internet access. All participants who met the above criteria and provided electronic informed consent were included in the sample.

Demographic characteristics, including age, gender, educational level, employment status, and marital status, were collected at baseline and reported in Table 1. Participants come from different regions of Northern Italy, such as Lombardy, Emilia-Romagna, Piemonte, and Veneto. The continuous variable (age) is reported as mean (M) and standard deviation (SD), whereas categorical variables (gender, education level, marital status, and employment status) are reported as frequencies and percentages. Differences between groups at baseline were analyzed with a Student* t*-test for age and a chi-square test for categorical data (Jamovi, Version 1.6.23.0) [56] and no significant differences emerged between groups (see Table 1).

**Table 1:** Participants’ characteristics

		*Experimental group (N* = *23)*	*Control group (N* = *26)*	*p*
*Age, Mean (SD)*		25.09 (2.31)	24.50 (2.27)	.375^a^
*Gender, N (%)*	Male	10 (43)	10 (38)	.721^b^
	Female	13 (57)	16 (62)	
*Education level (%)*	Senior high school	9 (39)	9 (35)	.221^b^
	Bachelor’s degree	4 (17)	10 (38)	
	Master’s degree or higher	10 (44)	7 (27)	
*Marital status (%)*	Single	13 (57)	12 (46)	.733^b^
	Partner	9 (39)	12 (46)	
	Cohabiting partner	1 (4)	2 (8)	
*Employment status (%)*	Unemployed	3 (13)	0	.166^b^
	Worker	13 (56)	13 (50)	
	Student	5 (22)	11 (42)	
	Student-worker or trainee	2 (9)	2 (8)	

^a^Based on independent sample Student* t*-test

^b^Based on chi-square test

### The study measures

Savoring beliefs and strategies were assessed both before and after the savoring intervention.

The Savoring Beliefs Inventory (SBI) was used to assess savoring beliefs [21]. Items from the original SBI were translated into Italian and then back translated by two independent translators. The outcome was discussed until all the translators agreed on reaching a consensus on cross-language equivalence. The SBI consists of 24 items that can be answered via a 7-point Likert scale ranging from 1 = *strongly disagree* to 7 = *strongly agree*. The SBI is composed of three subscales that, when added together, yield the total score (*α* = 0.89 for this study). The *anticipation* subscale (*α* = 0.74 for this study) measures how much a person believes he or she can savor through anticipation (e.g., “I feel a joy of anticipation when I think about upcoming good things”). The *present* subscale (*α* = 0.89 for this study) assesses how much the participant believes he or she can savor the present moment (e.g., “I know how to make the most of a good time”). The *reminiscence* subscale (*α* = 0.78 for this study) assesses how much the participant is perceived to be able to savor a memory (e.g., “I enjoy looking back on happy times from my past”). High scores on these subscales and the total scale indicate a greater perceived ability to savor positive events.

An adapted short version of the Ways of Savoring Checklist (WOSC) was used to assess savoring strategies [24]. The original WOSC was translated into Italian and then back translated by two independent translators. The outcome was discussed until all the translators agreed on reaching a consensus on cross-language equivalence. The original version is made up of ten sub-scales and a total of 60 items that can be responded to using a 7-point Likert scale from 1 = *strongly disagree* to 7 = *strongly agree*. We used an adapted short version of 14 items in this study, focusing only on a few amplifying strategies (*α* = 0.62 for this study), such as sharing with others (e.g., “I looked for people to share it with”), self-congratulation (e.g., “I told myself why I deserved this good thing”), absorption (e.g., “I thought only about the present—got absorbed in the moment”), and counting blessings (e.g., “I thought about what a lucky person I am that so many good things have happened to me”). Both before and after the intervention, we assessed both dimensions of subjective well-being: life satisfaction and negative and positive affect.

The Italian version of the Satisfaction With Life Scale (SWLS) was used to assess the cognitive dimension of subjective well-being and global life satisfaction (five items with responses on a 7-point Likert scale ranging from 1 = *strongly disagree* to 7 = *strongly agree*, (e.g., “In most ways my life is close to my ideal”) [57, 58]. The sum of all items is used to calculate the score, and high scores indicate high levels of life satisfaction (*α* = 0.84 for this study).

The Italian version of the Scale of Positive and Negative Experiences (SPANE) was used to measure positive and negative affect [59, 60]. This self-report scale consists of 12 items and is composed of two subscales: the positive affect subscale (6 items: positive, good, pleasant, happy, joyful, contented) (SPANE-P, *α* = 0.85 for this study) and the second negative affect subscale (6 items: negative, bad, unpleasant, sad, afraid, angry) (SPANE-N, *α* = 0.76 for this study). For all items, the response scale was a 5-point Likert scale (1 = *very rarely or never* and 5 = *very often or always*). The sum of positive and negative emotions can be used to calculate the score.

Furthermore, at the end of the first five activities, we asked participants to write down their thoughts about the exercise they had just performed. This was done after the fifth rather than the last activity because, at this point, participants were told that they would be sent a certificate of participation and access to the website for a full year as a gift for their participation. In this case, self-report measures would not have been appropriate: delivering gifts and then asking participants how they felt could in fact make them suspicious of the researchers’ intentions to manipulate their mood, and this, in turn, could dissipate the positive emotion induced [61, 62].

Finally, at the end of the intervention, the User Experience Questionnaire (UEQ) was used to investigate the participants’ experiences in using the online platform and doing the savoring exercises [63]. It contains six scales and is made up of 26 total items: *attractiveness* (*α* = 0.83 for this study) refers to the general impression with respect to the product (e.g., annoying—enjoyable), *efficiency* (*α* = 0.58 for this study) focuses instead on the organization and practicality of the website (e.g., impractical—practical), and *perspicuity* (*α* = 0.74 for this study) refers to the perceived ease of use (e.g., not understandable—understandable). Furthermore, *stimulation* (*α* = 0.85 for this study) refers to the interest and enthusiasm generated by its use (e.g., boring—exciting), and *novelty* (*α* = 0.81 for this study) focuses on its innovation and creativity (e.g., conservative—innovative). We have not included *dependability*, which focuses on how secure and predictable the interaction with the site was, because the internal consistency of the items in the scale appeared unacceptable (*α* < 0.5). The UEQ response modality is characterized by semantic differentials, and each item can be assigned a score between -3 and + 3, where -3 represents the most negative answer, 0 a neutral answer, and + 3 the most positive answer. For each scale, values between -0.8 and 0.8 represent a neutral evaluation, scores greater than 0.8 indicate a positive evaluation, and scores lower than -0.8 indicate a negative evaluation, while values near + 2 represent a very positive, near-optimal impression of participants [64].

A final item, with responses made on a 5-step Likert scale (1 = *very little* and 5 = *very much*), was used to assess the perceived usefulness of the intervention (“Referring to the entire intervention, how much do you think it was useful for you?”). Finally, participants made comments about the whole intervention, as previously mentioned.

### E-savoring intervention

**Fig. 1:**
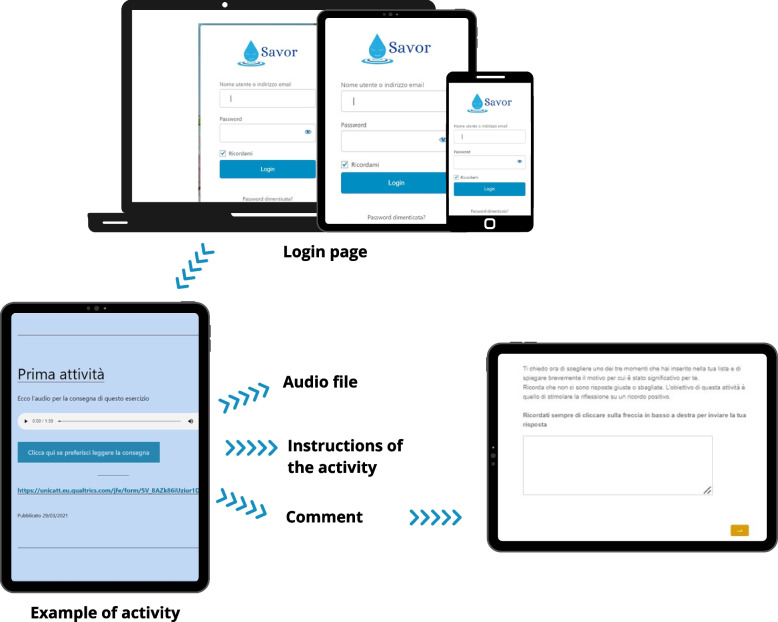
Online intervention: home page and example of activity

The entire intervention was designed and developed exclusively online to reach emerging adults in total safety during the COVID-19 pandemic. An ad hoc website was therefore designed and created via the WordPress platform (at the domain www.progettosavor.it), accessible from any device (smartphone, tablet, or computer) with an Android, iOS, Windows, or macOS operating system. The goal was to create a website with simple graphics that was intuitive and usable. Participants accessed the site with a username and password, and, once participants entered the website, they could access the online questionnaires (through a link to Qualtrics) and the intervention exercises (Table 2). Specifically, participants had to complete 2 exercises a week for this 3-week intervention, for a total of 6 exercises. Participants were emailed a reminder when each exercise was published on the website. Each exercise took about 10 min to complete, and participants were given the opportunity to access the exercise in either recorded audio or written text format, to give participants the chance to choose their favorite modality (Fig. 1).

**Table 2:** Savoring activities

*Temporal orientation*	*Exercise*	*Description*
*Past*	Recall of three positive past moments	Participants were invited to draw up a list of 3 happy moments that could also include important goals achieved in their life. This activity was inspired by a study by Lyubomirsky et al. [65]
*Past*	Recall and amplification of a positive past experience	Participants were asked to relive a positive experience
*Present*	Three good daily things to be thankful for	Participants were invited to identify, over the course of the day, 3 good things which they were grateful for. This activity was inspired by a study by Eamons and Mc Cullough [66]
*Present*	Absorption during an activity	Participants were asked to perform an activity that they particularly enjoyed and to pay attention to the perceptual experience related to positive emotions
*Future*	Anticipation of positive events	Participants were asked to imagine 3 positive events that could happen in the next few days and to pay attention to the emotions they felt while doing so. This activity was inspired by a study by Quoidbach et al. [67]
*Future and conclusion*	Boosting savoring activities and waiting for a gift	Participants watched a 5-min relaxation video in which music and natural scenarios were integrated. A narrative voice guided throughout the completed exercises to boost their effects

### Procedure

The study was conducted from October 2021 to February 2022, during the fourth wave of the COVID-19 pandemic in Italy. This pilot randomized controlled study used a between-subjects design and included two assessment moments. An email was sent to each participant interested in participating that provided information regarding the intervention. Personal credentials for accessing the website were sent to the experimental group participants. Participants were then invited to fill out informed consent forms and online baseline questionnaires—the SBI, WOSC Amplifying, SWLS, and SPANE (T0). The e-savoring intervention took place during the following 3 weeks. At the end of the intervention (T1), participants completed the same questionnaires—SBI, WOSC Amplifying, SWLS, and SPANE—with the integration of the UEQ and usefulness ad hoc questions only for the experimental group. The control group didn’t carry out any activity and completed only the pre- and post-intervention questionnaires in the same time frame as the experimental group. At T1, the control group received by email the credentials for accessing the e-savoring intervention for one year.

### Data analyses

All statistical analyses were conducted using Jamovi (Version 1.6.23.0) [56]. Group differences regarding psychological dimensions (savoring beliefs and strategies and subjective well-being) were examined at baseline using the Student *t*-test for independent samples. The intervention’s effect was examined through a repeated measures ANOVA with time as a within-subjects factor and group as a between-subjects factor. We were interested in the time x group interaction, which would indicate a differential change due to the intervention.

Qualitative data were analyzed thematically [68]. A thematic analysis identifies, analyses and reports patterns of meaning (themes) across the dataset, in order to answer the research question. This approach enabled concise analysis of a large amount of textual data by using a deductive approach in which unexpected themes could arise “bottom-up” from the data. Relevant parts of the data were systematically identified, before being coded into potential themes that were refined, defined, and named. Finally, a narrative analysis of the themes was conducted.

## Results

Before analyzing effects related to the e-savoring intervention, we compared the psychological dimensions at baseline and no differences emerged between groups (Table 3).

**Table 3:** Psychological dimensions at baseline

		*Range (min–max)*	*Experimental group (N* = *23)*	*Control group (N* = *26)*	*p*
*SBI, mean (SD)*	Anticipation	8–56	38.48 (7.35)	38.31 (6.65)	.932^a^
	Present	8–56	33.26 (8.19)	33.65 (9.19)	.181^a^
	Reminiscence	8–56	41.83 (5.23)	38.58 (7.54)	.090^a^
	SBI total	24–168	113.57 (16.43)	113.54 (19.88)	.996^a^
*SWLS, mean (SD)*		5–35	20.87 (6.31)	21.23 (6.05)	.839^a^
*SPANE, mean (SD)*	SPANE-P	6–30	18.65 (4.41)	19.69 (4.15)	.400^a^
	SPANE-N	6–30	15.04 (5.27)	14.92 (3.69)	.926^a^
*WOSC, mean (SD)*	Amplifying	14–98	61.74 (8.35)	60.23 (9.27)	.555^a^

*SBI* Savoring Belief Inventory, *SWLS* Satisfaction With Life Scale, *SPANE-P* Scale of Positive and Negative Experience – Positive, *SPANE-N* Scale of Positive and Negative Experience – Negative, *WOSC* Ways Of Savoring Checklist

^a^Based on independent sample Student *t-*test

### Effectiveness of the e-savoring intervention: preliminary results

A repeated measures ANOVA with time as a within-subjects factor and group as a between-subjects factor was performed for all variables (Table 4). We were interested in the time x group interaction, which would indicate a differential change due to the intervention. Descriptive data are shown as mean (M) and standard deviation (SD).

**Table 4:** Comparison between groups: repeated measures ANOVA interactions effects

		*Experimental group M (SD)*	*Control group M (SD)*	*Time x group, interaction effect*		
				*f*	*p*	*η*^*2*^
*SBI Anticipation*	Baseline	38.48 (7.35)	38.31 (6.65)	8.26	.006^a^	.02
	Post	41.78 (5.13)	37.81 (8.51)			
*SBI Present*	Baseline	33.26 (8.19)	36.65 (9.19)	4.59	.037^a^	.01
	Post	36.57 (7.09)	36.42 (10.16)			
*SBI Reminiscence*	Baseline	41.83 (5.23)	38.58 (5.74)	1.71	.197	.01
	Post	44.57 (5.29)	39.31 (7.64)			
*SBI Total*	Baseline	113.57 (16.43)	113.54 (19.88)	6.84	.012^a^	.02
	Post	122.91 (14.27)	113.54 (22.38)			
*SWLS*	Baseline	20.87 (6.31)	21.23 (6.05)	3.10	.085	.01
	Post	23.04 (5.86)	20.81 (7.14)			
*SPANE-P*	Baseline	19.65 (4.41)	19.69 (4.15)	6.40	.015^a^	.02
	Post	21.83 (4.28)	20.15 (4.92)			
*SPANE-N*	Baseline	15.04 (5.27)	14.92 (3.69)	0.51	.478	.00
	Post	13.96 (4.61)	14.58 (3.96)			
*WOSC Amplifying*	Baseline	61.74 (8.35)	60.23 (9.27)	3.68	.061	.01
	Post	66.35 (9.49)	61.12 (10.75)			

*M* mean, *SD* standard deviation, *SBI* Savoring Belief Inventory, *SWLS* Satisfaction With Life Scale, *SPANE-P* Scale of Positive and Negative Experience – Positive, *SPANE-N* Scale of Positive and Negative Experience – Negative, *WOSC Amplifying* Ways Of Savoring Checklist Amplifying

^a^Significant (*p* < 0.05)

Concerning the effects of the intervention on savoring beliefs (SBI), a significant group x time interaction was found. For the experimental group, there was a significant greater increase in savoring beliefs (total score) compared to baseline, and in particular in savoring the present (*p* < 0.05) and the future (*p* < 0.01), than in the control group, while no significant effect related to reminiscence emerged. Concerning the subjective well-being assessment, we did not find a significant change related to its cognitive dimension, such as life satisfaction (SWLS), but we found a significant change related to its affective dimension (*p* < 0.05), in particular for the positive emotions (SPANE-P): the experimental group showed a significantly greater increase in the positive emotions compared to the control group, while no significant changes about the negative emotions (SPANE-N) were found. Moreover, the analysis of scores obtained by the participants in the adoption of savoring strategies (WOSC Amplifying), including sharing with others, self-congratulation, absorption, and counting blessings, showed a tendency toward a significant increase (*p* = 0.061) in the experimental group compared to the control group.

### Qualitative analysis of visualized experiences

Qualitative data were thematically analyzed in order to explore the research question: What were participants' emotional experiences activated by the savoring exercises? There was no missing data, because the online questionnaire required participants to provide qualitative comments about each activity performed. Two principal themes were identified: a) activated positive emotions [69, 70] and experienced bodily awareness, and b) the content of the visualized experience. As far as the content is concerned, we considered the social dimension (*shared experiences,* related to activities performed together with others such as friends, relatives, partner, etc. vs. *individual activities* that take place alone), the scenario (*natural environments* referred to outdoor natural places vs *artificial environments* referred to indoor places such as offices, homes, gyms, etc.), and the performed activity (*occupational activities,* such as work and/or study, vs. *recreational activities,* such as reading, walking, and other leisure activities). Details are reported in Table 5.

**Table 5:** Qualitative evaluation of the visualized experiences: principal themes (number of participants)

		*Recall three positive past moments*	*Recall and amplify a positive past experience*	*Three good daily things to be thankful for*	*Absorption during an activity*	*Anticipation of positive events*
*Positive emotions*	Happiness	7	5	8	2	10
	Joy	2	12	2	2	12
	Enthusiasm	1	2	1	0	8
	Gratitude	5	4	13	3	7
	Pride	9	2	4	7	6
	Love	2	1	2	0	1
	Hope	0	1	1	1	6
	Serenity	5	6	5	12	8
*Bodily awareness*	Bodily Awareness	0	10	0	5	3
*Experience content*	Shared Experiences	15	8	19	4	21
	Individual Experiences	11	8	9	22	13
	Natural Environments	4	4	6	8	7
	Artificial Environments	1	2	4	4	4
	Occupational Activities	10	5	3	0	5
	Recreational Activities	9	6	12	22	21

The first activity, consisting of asking participants to list three happy moments, led participants to recall both shared (65%) and individual experiences (48%), and mostly related to occupational activities (43%). These memories elicited positive emotions, and in particular pride and satisfaction (39%) and happiness (30%). The second activity, consisting of recalling and amplifying one positive moment, led participants to recall both shared and individual experiences (both 35%) and related to both occupational and recreational activities (22% and 26%, respectively). This activity mostly elicited joy (52%) and boosted bodily awareness (43%). The third activity, asking participants to identify three daily things to be grateful for, led them to visualize mostly shared experiences (83%) related to recreational activities (52%) and also elicited gratitude (56%) and happiness (35%). The fourth activity, which asked participants to be fully involved in the enjoyable activity they were doing, led participants to visualize mostly individual experiences (96%) performed in a natural environment (35%) during recreational time (96%). This activity elicited serenity (52%) and pride and satisfaction (30%). The fifth activity, asking participants to imagine at least two positive events that could happen in the next few days and pay attention to the emotions aroused by the imagined events, led participants to anticipate both shared (91%) and individual experiences (56%), mainly related to natural environments (30%) and recreational activities (91%), which mostly elicited happiness (43%), enthusiasm (35%), and gratitude (30%).

### User experience and perceived usefulness

Furthermore, at the end of the e-savoring intervention, participants filled out the User Experience Questionnaire. Participants reported a very positive evaluation of the system in terms of *perspicuity, attractiveness,* and *efficiency*; *stimulation* and *novelty* were also positively evaluated. Furthermore, participants evaluated the intervention as useful (the average rating is between "quite useful" and "very useful," median = 4). Details are reported in Table 6.

**Table 6:** E-savoring intervention evaluation: user experience

	*M*	*SD*	*Positive cut-off*	*Range*
*Attractiveness*	1.88	0.73	> 0.8	-3/ + 3
*Perspicuity*	2.41	0.76	> 0.8	-3/ + 3
*Efficiency*	1.90	0.65	> 0.8	-3/ + 3
*Stimulation*	1.54	1.05	> 0.8	-3/ + 3
*Novelty*	1.04	1.07	> 0.8	-3/ + 3
*Perceived usefulness*	3.61	0.78	n.a	1–5

*M* mean, *SD* standard deviation

Regarding perceived usefulness, a qualitative analysis was also conducted. A descriptive analysis of the participants’ comments about the overall evaluation of the intervention revealed some key areas: 15 out of 23 participants left a written comment; 5 out of 15 participants (33%) reported increased awareness of positive experiences and associated feelings. For example, a 24-year-old woman stated, “This path has been a path of inner reflection that helps you reflect on your reactions to the moments of your life. […] For me, it was interesting and enjoyable to take this type of path. It was useful to stop and think and to understand yourself a little more.” Furthermore, 7 out of 15 participants (47%) reported that they had learned strategies to boost positive emotions. For instance, a 26-year-old woman asserted, “I found this to be a very clear method of learning a simple but sometimes difficult concept without input. It gave me the opportunity to reflect, and stop, and think about the good things, which exist and are considerably more common than the bad ones. I will treasure these exercises, especially in times when everything seems difficult. Thank you.” Moreover, 8 out of 15 participants (53%) expressed their intention to put into practice the strategies they learned in the future. For example, a 23-year-old woman reported, “It helped me especially in reliving the positive emotions of past events. I was really savoring all the emotions I felt. In the second activity, I relived the happy moment in my mind, and it really felt like I was there, and I felt a lot of the happiness I felt in that event. I had never tried to do such a thing; I had never been able to savor so well the feelings from a past event. […] I've found that I can relive those emotions whenever I want thanks to this activity! I think I will take all the activities of this project with me for the rest of my life. This project […] has been a great practical help! Thank you!".

## Discussion

The COVID-19 pandemic has proven to be a challenging time for emerging adults who reported a worrying decline in mental health and subjective well-being [5, 71]. Starting from these premises, the present study developed and investigated the effects of a self-help e-savoring intervention in improving savoring beliefs and strategies and the subjective well-being of emerging adults.

Findings partially confirmed our hypotheses. Concerning the effectiveness of the intervention on increasing savoring beliefs (H1), participants reported significant improvements and this result is in line with other studies reporting an increase in total SBI levels [49]. Participants felt more able to savor both upcoming positive events and the present moment. Participants showed a slight but not significant improvement in reminiscence beliefs. These results are in line with Bryant’s [21] statement that people typically consider themselves better at savoring through reminiscence. Furthermore, before the intervention (baseline), reminiscence levels were higher than at the other times; thus, participants already felt competent to savor memories and positive past experiences.

Concerning the intervention’s effect on SWB, the second hypothesis (H2) was partially confirmed. On the one hand, there was not a significant increase in life satisfaction, probably due to the short duration of the intervention. The brevity of this intervention (6 activities in 3 weeks) allowed us to achieve a very good adherence rate but did not sustain a change in the global cognitive assessment of one's life. More time is needed to achieve a sustained change—which was reported in other studies that had longer-lasting interventions (e.g., 12 weeks) [35, 57]. In addition, other studies that integrated different positive psychology strategies (e.g., mindfulness, gratitude, optimism, strengths, etc.) or more savoring strategies found increases in life satisfaction as well as other positive psychological outcomes [47, 72, 73]. On the other hand, the intervention was effective in increasing positive emotions. The opportunity to write comments about the performed savoring activities may have stimulated participants' reflection. Indeed, writing about positive experiences can promote the building of a logical narrative, which in turn fosters the maintenance of positive emotions [50]. In line with the study carried out by Yu et al. [50], we did not find a change in negative emotions. It is important to highlight that savoring does not exclusively include focusing on the positive aspects of life while ignoring the negative ones. Indeed, the benefits of savoring are visible in the ability to notice and appreciate positive moments despite and during life difficulties [25, 74]. Thus, in our study, we cannot exclude the possibility that some negative emotions related to the challenging situation of the pandemic affected participants’ emotional states.

Regarding the effectiveness of the intervention in increasing savoring strategies as a research question, participants reported a tendency toward a significant improvement. Interestingly, after a 3-week intervention, a change in participants’ savoring behaviors was triggered. Nevertheless, it is not possible to compare this result with those of previous studies because, to our knowledge, no savoring interventions have investigated the effects on savoring strategies [75, 76]. Future studies that assess this dimension are needed.

Qualitative analyses revealed that participants experienced several positive emotions during the visualized events, such as happiness, joy, pride, gratitude, and serenity. While they were more absorbed by individual activities in savoring the present moment, they were oriented toward savoring relational activities in the future. This finding highlights the importance of encouraging positive moments with family, friends, and partners after social distancing ends. Recreational visualizations prevailed over work activities. Due to the importance and need of promoting well-being and positive emotions in the workplace, savoring interventions could be adapted to this context [77].

Regarding the user experience, the website was perceived as easy to use. Indeed, it was designed and created specifically for this intervention, and all functions were intuitive. In addition, most participants perceived the whole intervention as useful, as the savoring activities elicited positive emotions and reflections.

Taking into account the present study’s findings as a whole, it seems that the online savoring intervention represents an effective protocol to sustain emerging adults’ subjective perception of being able to intensify positive emotions related to the present and the future and to boost positive affect during the COVID-19 pandemic. The present findings are consistent with the positive outcomes reported by other previous online savoring interventions based on psychoeducational resources or proposing different strategies [49, 50].

### Limitations and strengths

The current study focuses on the development, implementation, and preliminary evaluation of a new online savoring intervention with emerging adults during the COVID-19 pandemic, leaving the door open for future research to address its limitations and refine its findings.

First, we included a control group that did not engage in any activity other than completing the assessments at the two times, and, according to Kraiss et al. [78], this issue could have an impact on the evaluation of intervention effectiveness. This limitation requires us to view the results with caution and encourage future studies to test the intervention with active control groups. Second, because we used convenience and snowball sampling methods, we have to recognize that this choice could have an influence on the results’ generalizability. Third, we used savoring scales not validated in Italian. On one hand, the ad hoc version of the WOSC was not fully reliable; on the other hand, even if the SBI had not previously been validated in Italian, the reliability of the subscales was good. Furthermore, these results can be considered preliminary and only partially generalizable, as they involve a small sample of emerging adults from Northern Italy.

Despite these limitations, we can recognize some strengths of this study. One is the implementation of a self-help savoring intervention, solidly anchored in some of the amplification strategies proposed by Bryant and Veroff [24]. Furthermore, the online implementation of the intervention can be viewed as one of its strengths, as it allowed participation of emerging adults from different regions during a time when fear of contagion was still high and young people struggled to resume the normal activities they practiced before the pandemic. The ability to amplify positive emotional experiences anchored in one's personal history allows one to feel highly engaged in the activities, and the ease of the exercises fits well with the self-help approach.

Finally, the high adherence rate represents another strength of the current study, probably sustained by engaging activities proposed in a short intervention. In other cases, extended online savoring interventions had a high dropout rate due to the significant effort and time required [49].

## Conclusions and implications

The online self-help e-savoring intervention showed great potential for promoting well-being and savoring in the emerging adult population during the COVID-19 pandemic. The high level of adherence and the appreciation for the intervention led us to speculate that it could be adapted and tested with a large sample of participants of various ages. Furthermore, future studies are encouraged in assessing the maintenance of achieved results with a follow-up evaluation. Finally, given the increase in mental disorders such as anxiety and depression in emerging adults as a result of the COVID-19 pandemic, as well as recent research on the role of savoring in reducing depression, it might be worthwhile to apply this intervention to this population as well as others affected by these mental health conditions [79].

## Data Availability

The dataset used and analysed during the current study are available from the corresponding author on reasonable request.

## Abbreviations

SWB: Subjective Well-Being
ANOVA: Analysis of Variance
ISTAT: Istituto Nazionale di Statistica
PPIs: Positive Psychology Interventions
OPPIs: Online Positive Psychology Interventions
M: Mean
SD: Standard Deviation
SBI: Savoring Beliefs Inventory
WOSC: Ways of Savoring Checklist
SWLS: Satisfaction With Life Scale
SPANE: Scale of Positive and Negative Experiences
UEQ: User Experience Questionnaire
